# Exceptional Eight-year Response to Stereotactic Radiosurgery Monotherapy for Multiple Brain Metastases

**DOI:** 10.7759/cureus.2001

**Published:** 2017-12-29

**Authors:** Ronny Kalash, Phillip M Pifer, Sushil Beriwal, Scott M Glaser, John A Vargo, Dwight E Heron

**Affiliations:** 1 Department of Radiation Oncology, University of Pittsburgh Cancer Institute, UPMC; 2 Department of Radiation Oncology, West Virginia University School of Medicine/Ruby Memorial Hospital; 3 Radiation Oncology, City of Hope, Duarte, Ca, Usa

**Keywords:** brain metastasis, truebeam, stereotactic radiosurgery, metastatic breast cancer, cyberknife™, gamma knife radiosurgery

## Abstract

Breast cancer represents the second leading cause of brain metastases in women. Once diagnosed, brain metastases have been associated with a rapidly progressive and universally poor prognosis. Breast cancer patients, particularly those with advantageous disease characteristics, may achieve extended survival. This extended life expectancy highlights the importance of effective intracranial treatments that minimize treatment-related late toxicity.

Whole brain radiation therapy (WBRT) remains a standard of care palliative option; however, concerns remain regarding the late neurocognitive effects. Stereotactic radiosurgery (SRS) provides dose-escalated radiation therapy over a shortened course, maintaining equivalent survival and minimizing normal brain tissue exposure.

Herein, we present a breast cancer patient who demonstrated an exceptional response and remained functionally independent following 12 SRS courses targeting 14 unique brain metastases over eight years. The case illustrates the efficacy of SRS alone, as well as the comparable utility of multiple SRS treatment techniques (Gamma Knife (AB Elekta, Stockholm, Sweden), CyberKnife (Accuray, Sunnyvale, California), and TrueBeam (Varian Medical Systems, Palo Alto, California)).

## Introduction

Breast cancer remains the most commonly diagnosed malignancy and the second leading cause of brain metastasis in women [1-2]. Historically, the presence of brain metastases has been associated with a universally poor prognosis. However, breast cancer patients represent a unique cohort in which factors such as phenotype, performance status, age, and extracranial disease burden may portend an extended survival. As highlighted in the validated disease-specific graded prognostic assessment (DS-GPA), favorable subsets with HER2+/Luminal B breast cancer have a median survival of 25-27 months [3]. This extended life expectancy highlights the importance of effective intracranial treatments that minimize treatment-related late toxicity.

Whole brain radiation therapy (WBRT) has routinely been used to promote intracranial control and palliate the symptoms of brain metastases. However, WBRT has been associated with risks of significant late neurocognitive decline, which can detract from the quality of life, especially in long-term survivors. Stereotactic radiosurgery (SRS) allows the delivery of highly conformal dose-escalated radiation therapy over a short time course while minimizing normal brain tissue exposure. SRS decreases the likelihood of a decline in learning and memory function as compared to WBRT while maintaining equivalent survival [4-5]. Currently, multiple SRS delivery platforms are available including Gamma Knife, linear accelerator-based therapies, and proton beam therapy. Herein, we present a breast cancer patient with multiple brain metastases with an exceptional eight-year response to SRS alone across several treatment platforms, demonstrating the efficacy of SRS alone as well as the comparable utility of multiple SRS treatment techniques (Gamma Knife (AB Elekta, Stockholm, Sweden), CyberKnife (Accuray, Sunnyvale, California), and TrueBeam (Varian Medical Systems, Palo Alto, California)).

## Case presentation

A 53-year-old woman presented with a self-palpated, biopsy-proven, invasive ductal carcinoma (estrogen/progesterone receptor positive, HER-2/neu positive) of the left breast in December 2004. She underwent neoadjuvant docetaxel/cyclophosphamide chemotherapy followed by a total mastectomy and axillary node dissection in May 2005, with pathology demonstrating ypT1N1M0 Stage IIA grade three invasive ductal carcinoma. She was subsequently treated with anastrozole and trastuzumab until three symptomatic brain metastases were discovered in June 2006. No additional distant metastases were evident. Her systemic therapy from that point included lapatinib and tamoxifen until 2010 and fulvestrant thereafter. 

**Figure 1:**
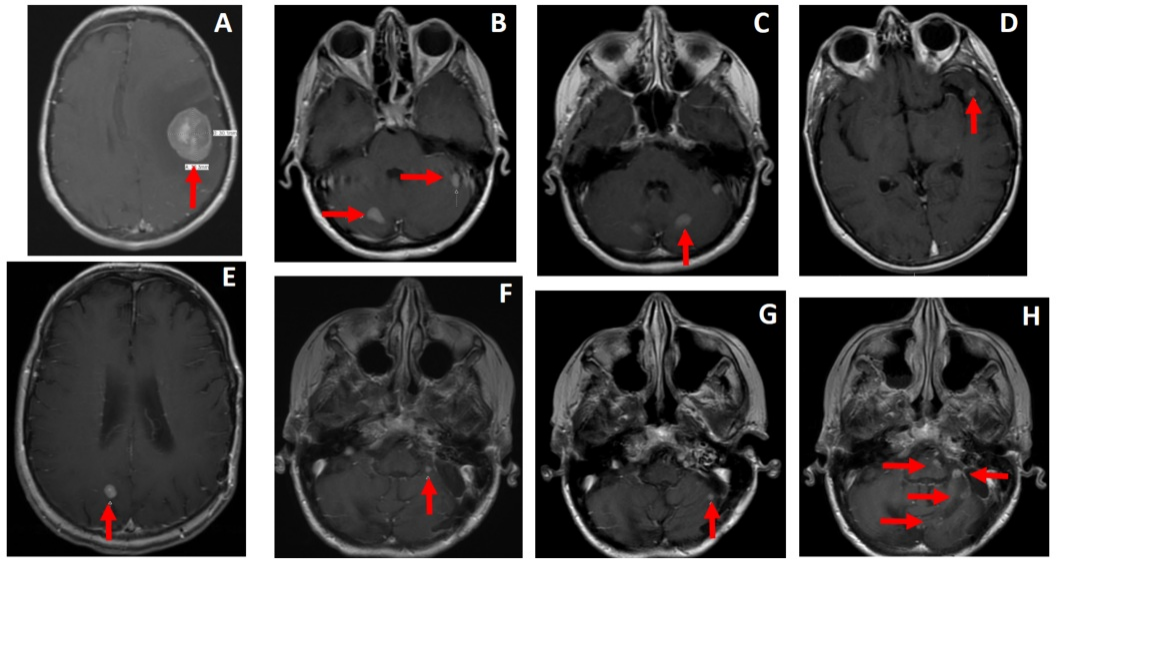
Pre-SRS treatment T1-weighted post-contrast brain MRI demonstrating intracranial metastasis Brain metastasis presenting time points include initial presentation 06/2006 (A&B), 03/2008 (C), 07/2009 (D), 02/2013 (E), 12/2013 (F), 11/2014 (G). Window (H) represents multifocal progression prior to the initiation of WBRT in 03/2015. MRI: magnetic resonance imaging; SRS: stereotactic radiosurgery; WBRT: whole brain radiation therapy.

**Figure 2:**
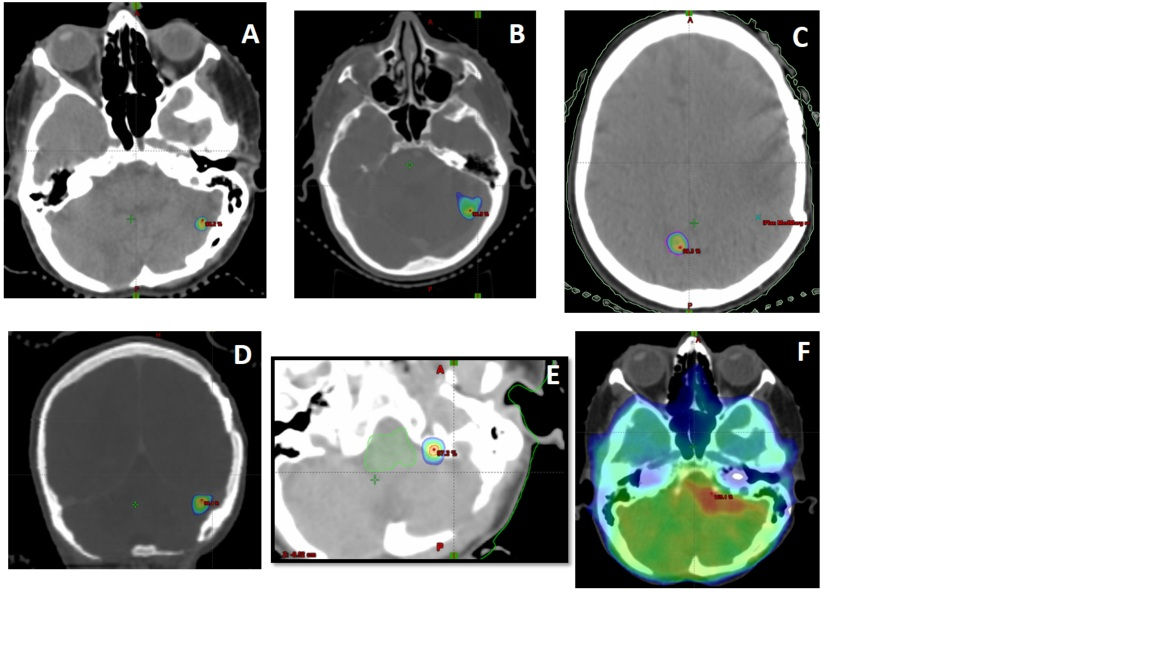
SRS treatment fields The 80% isodose line of SRS treatments delivered on 06/2006 (A), 11/2014 (B), 02/2013 (C), 03/2008 (D), 12/2013 (E). The whole brain course with SIB to gross disease delivered in 03/2015 is depicted in window (F). SRS: stereotactic radiosurgery; SIB: synchronous integrated boost.

Over her eight-year experience with brain metastases, she received multiple courses of SRS, as outlined in Table 1. Pre-treatment magnetic resonance imaging (MRI) scans, as well as treatment fields, can be seen in Figure 1 and Figure 2. Briefly, the patient underwent surgical resection of an index left frontotemporal lesion and subsequent Gamma Knife (GK) radiosurgery to each of three metastatic sites in June 2006. A new left Sylvian fissure lesion and bilateral cerebellar lesions were treated with CyberKnife radiosurgery (CKRS) in July 2007 and March 2008, respectively. In July 2009, a new left Sylvian fissure was treated with CKRS, and in November 2010, this lesion gradually increased in size and was re-treated with CKRS. A new right parietal lobe lesion was treated with CKRS in February 2013. Three left cerebellum lesions were treated with Truebeam in December 2013. In May 2014, a new left lateral cerebellar lesion was treated with CKRS. In November 2014, the local progression of the left lateral cerebellum lesion was treated with TrueBeam radiosurgery.

**Table 1:** Summary of radiation treatment from 2006 to 2015 FX: fraction; SIB: synchronous integrated boost; IMRT: intensity-modulated radiation therapy; Lt: left; Rt: right.

Date	Treatment Site	Dose	Technique	Volume (cc)
6/15/2006	Post Op Cav + 2 Lesions	18Gy/1Fx	GammaKnife	5.20, 0.46 , 0.25
8/10/2007	Lt Sylvian Fissure	24Gy/1Fx	CyberKnife	0.51
3/27/2008	Lt Medial Cerebellar	18Gy/1Fx	CyberKnife	0.73
3/27/2008	Rt Medial Cerebellar	21Gy/1Fx	CyberKnife	1.41
7/9/2009	Lt Sylvian Fissure	24Gy/1Fx	CyberKnife	0.84
11/22/2010	Lt Lateral Cerebellar	20Gy/1Fx	CyberKnife	0.46
2/26/2013	Rt Parietal	21Gy/1Fx	CyberKnife	0.61
12/12/2013	Lt Posterior Cerebellar	18Gy/1Fx	TrueBeam	0.81
12/12/2013	Lt Medial Cerebellar	18Gy/1Fx	TrueBeam	0.32
12/12/2013	Lt Anterior Cerebellar	18Gy/1Fx	TrueBeam	0.11
5/15/2014	Lt Lateral Cerebellar	24Gy/1Fx	CyberKnife	0.18
11/23/2014	Lt Lateral Cerebellar	24Gy/3Fx	TrueBeam	2.42
3/31/2015	Whole Brain	25Gy/10Fx , 40Gy SIB	IMRT	

In March 2015, she developed dysarthric speech and an MRI discovered progressive disease in the midbrain and right temporoparietal lobe, as well as enhancement within the posterior fossa, concerning for leptomeningeal disease. She, thus, underwent a 10-fraction course of hippocampal, sparing whole brain radiation therapy. She completed each course of SRS without treatment-related toxicity, a detriment to the quality of life, and remained neurologically intact until her final intracranial progression. Six months after receiving whole brain radiation therapy, she suffered a further progression of the disease and elected to pursue hospice care.

## Discussion

This case underscores the potential durable preservation of quality of life and disease control with the utilization of SRS in the treatment of a limited intracranial tumor burden. The patient underwent 12 SRS courses, targeting 14 lesions over eight years and remained functionally independent until systemic deterioration requiring hospice care. She tolerated each course of SRS without incident until eventually suffering progressive disease requiring WBRT. 

Although generally very well-tolerated, the toxicity associated with SRS may include edema, seizures, and radionecrosis. RTOG 90-05 demonstrated an increased rate of neurotoxicity with increasing tumor diameter, likely secondary to increased normal tissue dose [6]. This, in combination with the single-institution series, suggests that tumor volume, rather than the number of metastases, may serve as a better gauge of the outcome when considering SRS over WBRT [7]. The paradigm of volume rather than the number of metastases is well highlighted in the presented case, with each new presentation of an intracranial disease being of limited volume and, thus, requiring minimal normal brain tissue treatment, likely contributing to the overall exceptional outcome [8]. The total cumulative volume of disease/cavity treated by the prescription dose was 14.3 cc, yielding a low volume of normal brain tissue receiving greater than 12Gy.

GK SRS has long been a gold standard for intracranial SRS, and linear accelerator-based systems have since made significant progress. This patient received treatment via GK-SRS as well as the CyberKnife and TrueBeam platforms. Population studies demonstrate that the use of Linac-based SRS is more frequent in community settings when a cancer facility is less than 20 miles away [9]. No consistent differences in clinical efficacy have been reported between SRS platforms to date. Comparative studies have reported the shortest beam-on time with TrueBeam, and the lowest peripheral normal brain tissue dose with GK-SRS [10]. This may provide a theoretical benefit in the safety of treatment delivery and normal tissue dose. This study includes a single patient experience of each major treatment platform (GK-SRS, CKRS, and TrueBeam STX with BrainLAB), each demonstrating an excellent long-term outcome.

This patient exemplifies the excellent long-term outcome possible with SRS treatment of metastatic breast cancer. As the efficacy of systemic therapy continues to improve, the importance of SRS in the management of brain metastases will continue to grow. Currently, SRS provides efficient, effective, and less-toxic treatment of intracranial disease to promote the most desirable, long-term outcome. On-going trials will reveal the added efficacy and safety of SRS alongside systemic agents with the penetration of the blood-brain barrier.

## Conclusions

SRS represents an effective treatment modality to control intracranial metastases. Multiple treatment platforms allow exceptional and equivalent long-term outcomes and may provide an improved long-term quality of life compared to whole brain radiation therapy.
